# A next‐generation vaccine for broader and long‐lasting COVID‐19 protection

**DOI:** 10.1002/mco2.138

**Published:** 2022-05-02

**Authors:** Jinxiang Xi, Xiuhua April Si

**Affiliations:** ^1^ Department of Biomedical Engineering University of Massachusetts Lowell Massachusetts USA; ^2^ Department of Aerospace Industrial, and Mechanical Engineering California Baptist University Riverside California USA

**Keywords:** adenoviral vectored vaccine, alveolar macrophage, intramuscular, intranasal, mucosal immunity, next‐generation COVID‐19 vaccine

Recently, Afkhami et al. (Cell, 185: 896–915) presented an interesting study that demonstrated equivalent or better immunity from intranasal vaccination compared to traditional intramuscular vaccination.^1^ This study shed light on many questions surrounding mucosa immunity induced by the adenoviral‐vectored trivalent vaccine, which is promising as an inhaled candidate for both short‐term and long‐term protections against coronavirus disease 2019 (COVID‐19). Up to date, approved COVID‐19 vaccines and boosters are all administered intramuscularly. Interests in developing mucosal vaccination have been arising again during this pandemic for its convenience and painless characteristics.^2^ COVID‐19 is known as respiratory infectious disease, and the severe acute respiratory syndrome coronavirus 2 (SARS‐CoV‐2) virus infects humans starting from the upper airway, particularly the nose or mouth. The elevated expression of angiotensin‐converting enzyme 2 (ACE2) protein and CD143 in the posterior nose make it the initial invading site of the virus, because of which it has been the nasal swab sampling site for COVID‐19 tests. Similarly, applying vaccines directly to the nasal mucosa has great potential to lower viral load inside the nasal airway, thus minimizing viral spreading to the lung, brain, and surrounding subjects.^3^ Moreover, intranasal vaccination via the respiratory mucosa can bypass the preexisting antivector immunity.^4^

Clinical trials of inhaled vaccines and therapeutics in the form of nasal sprays have been conducted using existing or modified vaccines, such as Carrageenan, Povidone‐Iodine, Ivermectin, Xylitol, etc.^5^ The recent study by Afkhami et al. demonstrated that mucosal immunization using Adenoviral‐vectored multivalent vaccines could be a convenient, cost‐efficient, and painless vaccination strategy to achieve adequate protection against COVID‐19 infections.^1^ Even a single‐dose intranasal application induced all‐around mucosal immunity against ancestral SARS‐CoV‐2 and variant strains. In the following sections, we will present the insights gleaned from the study of Afkhami et al. on the following questions.^1^ (1) Why can the new vaccine protect against SARS‐CoV‐2 ancestral virus and variants, but existing S1‐based vaccines cannot? (2) how does the new vaccination strategy work in terms of efficacy comparison between (a) intranasal versus intramuscular routes (Figure 1B), (b) system versus local, antigen‐specific immunity (Figure 1C–E), and (c) trivalent versus bivalent versus monovalent vaccines (Figure 1A).

**FIGURE 1:**
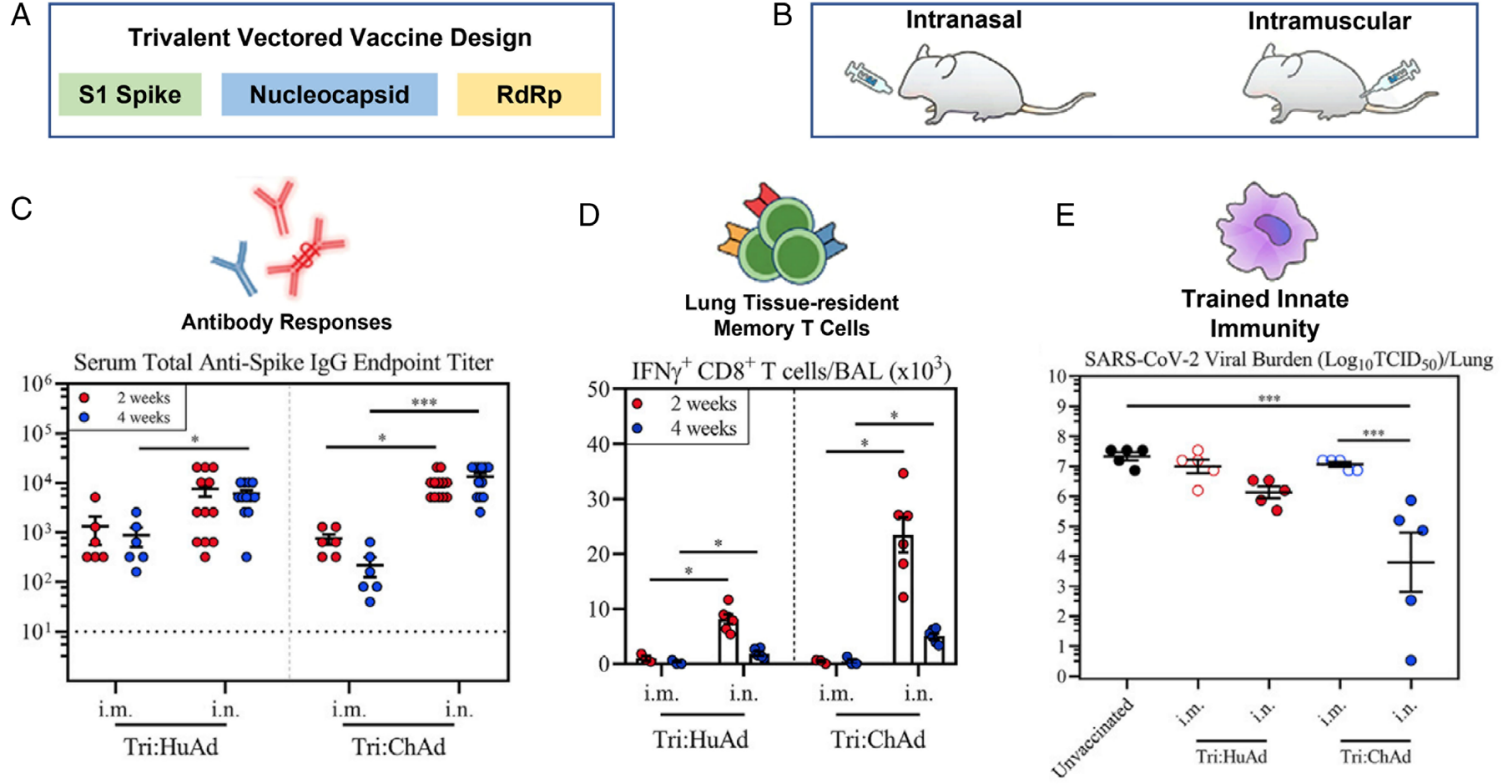
Design and test of a trivalent vectored vaccine: (A) trivalent vectored vaccine expressing three antigens: S1, nucleocapsid, and RdRp, (B) intranasal versus intramuscular vaccine delivery deposition pattern, (C) anti‐spike response, (D) T cell response (RdRp), and (E) trained innate immunity. Reprinted with permission^1^

First, why does the intranasally administered trivalent vaccine work better than conventional vaccination methods? Considering the mounting evidence that the protection provided by existing vaccines wanes with time and for emerging variants, to successfully develop an effective, long‐lasting vaccine that can protect against the ever‐evolving strains of SARS‐CoV‐2 is of utmost interest to all; but how will it work? To invade the cell and replicate itself, SARS‐CoV‐2 expresses a series of antigens to facilitate this process, such as the spike 1 and 2 proteins (S1, S2), nucleocapsid (N), and RNA‐dependent RNA polymerase (RdRp), among others. S1 is a transmembrane protein that can identify and tightly binds to the ACE2 on the cell surface via its receptor‐binding domain (RBD).^6^ The other antigen, S2, mediates the fusion between the viral and cellular membranes, while nucleocapsid binds to the RNA and acts on viral assembly. To replicate the viral RNA, enzyme RdRp is required, which accelerates DNA‐independent replication/transcription processes.^7^

Note that previous vaccine designs have concentrated on one antigen only, such as the spike protein. The newly developed vaccine by Afkhami et al. targeted three antigens at different stages of the viral invasion/replication (Figure 1A).^1^ In addition to the conventional S1, it also included the entire nucleocapsid and partial RdRp proteins to broaden T cell immunity against additional viral antigens. The nucleocapsid is the most common antigen in T cell epitopes in post‐COVID subjects.^8^ The nucleocapsid‐based vaccines have been demonstrated to elicit spike‐independent systemic immunity. T cells expressing nucleocapsid and RdRp are cross‐reactive with other coronaviruses.^9^ In Particular, RdRp has been considered as the Achilles heel of viral infection because it can be readily manipulated to interrupt the replication. Normally, RdRP catalyzes the addition of four different nucleotides in a prescribed sequence. However, when a ribonucleotide is inserted into the sequence, it leads to the termination of the replication process.^10^ As a result, including the N and RdRp antigens besides S1 was expected to elicit a broader and long‐lasting immunity.

Second, how well can it improve clinical outcomes and reduce the viral load? This question was answered from several aspects: anti‐spike protein humoral response, adaptive immunity in the airway, mucosal responses, and trained innate immunity (TII).

*Anti‐spike protein humoral response*: Considering the anti‐spike protein responses in the body fluids, single‐dose intranasal immunization performed better than the intramuscular route both systemically and locally in the lung. The anti‐spike and RBD‐specific IgG responses for the Tri:HuAd and Tri:ChAd vaccines in serum following the intranasal immunization were about 1 and 2 logs higher than intramuscular immunization, respectively (Figure 1C). In addition, the intranasal Tri:ChAd vaccine induced notable RBD‐specific B cells in the respiratory tract epithelium, while intramuscular vaccination failed to do so. These findings are significant because they presented evidence that even for the same vaccine, the intranasal route could be more effective than the intramuscular one.

*Adaptive immunity in the airway*: Robust adaptive immunity in the airway was elicited by the intranasal Tri:HuAd and Tri:ChAd vaccinations, as demonstrated by the significantly elevated magnitude of the antigen‐specific CD8^+^ T cells. By contrast, intramuscular immunization failed to induce measurable antigen‐specific CD8^+^ T cells in the respiratory tract, regardless of the viral vector (i.e., S1, nucleocapsid, and RdRp) (Figure 1D).

*Mucosal tissue‐resident memory cell responses and TII*: Figure 1E highlights that a single‐dose intranasal, not intramuscular, vaccination induced durable mucosal tissue‐resident memory (T_RM_) cell responses, as well as the TII. Mucosal T_RM_ cells play a crucial role in host defense, while airway macrophages (AMs) are the major innate immune cells fighting SARS‐CoV‐2, especially in early innate immune control. While intramuscular vaccinations hardly induced any class‐II major histocompatibility complex (MHC‐II, an index for the level of trained AM), intranasal immunization using Tri:ChAd yielded multiple populations with a high‐level expression of MHC II or CD11b in addition to the large cluster of AMs.

It is also interesting to compare the relative immunization efficacy between the trivalent‐vectored vaccine and its bivalent and monovalent counterparts to appreciate the benefits from the new multi‐vector‐based design. Considering the viral load, for a given dose of 1 × 10^5^ PFU SARS‐CoV‐2, severe clinical illnesses were observed in unvaccinated animals, while no viruses were detected with the trivalent vaccine. Bi‐valent (nucleocapsid and RdRp) decreased the viral load by 3 and 2 logs in the brain and lung, respectively. Considering clinical symptoms, comparable body weight losses were noted in unvaccinated and monovalent (S1) vaccinated animals. However, there was insignificant weight loss in both bivalent and trivalent vaccinated animals. Likewise, large‐scale pathology was observed in the lungs of the unvaccinated and monovalent vaccinated animals, while no apparent gross pathology was observed in bivalent and trivalent vaccinated animals. These results indicated that the inclusion of nucleocapsid/RdRp in the new vaccine design offered improved protective immunity and thus decreased the chance of viral spreading than conventional S‐1‐based vaccines.

In summary, by demonstrating the high efficacy of a trivalent vector‐based vaccine with single‐dose intranasal applications, this recent study by Afkhami et al. opened the door to the long‐waited needle‐free vaccination.^1^ With noninvasive and easy‐to‐apply features, better compliance and more frequent vaccinations are possible for the purposes of both long‐term and short‐term protections.

## CONFLICT OF INTEREST

The authors declare that they have no conflict of interest.

## AUTHOR CONTRIBUTIONS

JX and XS conducted the literature review, analyzed the data, and wrote the manuscript.

## ETHICS STATEMNET

Not applicable.

## Data Availability

The data used in this perspective are available from the corresponding author upon request.
